# Room temperature large-scale synthesis of layered frameworks as low-cost 4 V cathode materials for lithium ion batteries

**DOI:** 10.1038/srep16270

**Published:** 2015-11-23

**Authors:** A. Shahul Hameed, M. V. Reddy, M. Nagarathinam, Tomče Runčevski, Robert E Dinnebier, Stefan Adams, B. V. R. Chowdari, Jagadese J. Vittal

**Affiliations:** 1 Department of Chemistry, 3 Science drive 3, National University of Singapore, Singapore 117543; 2 Advanced batteries lab, Department of Physics, 2 Science drive 3, National University of Singapore, Singapore 117551; 3 Department of Materials Science and Engineering, 9 Engineering drive 1, National University of Singapore, Singapore 117575; 4 Max Planck Institute for Solid State Research, Heisenbergstrasse 1, 70569 Stuttgart, Germany

## Abstract

Li-ion batteries (LIBs) are considered as the best available technology to push forward the production of eco-friendly electric vehicles (EVs) and for the efficient utilization of renewable energy sources. Transformation from conventional vehicles to EVs are hindered by the high upfront price of the EVs and are mainly due to the high cost of LIBs. Hence, cost reduction of LIBs is one of the major strategies to bring forth the EVs to compete in the market with their gasoline counterparts. In our attempt to produce cheaper high-performance cathode materials for LIBs, an rGO/MOPOF (reduced graphene oxide/Metal-Organic Phosphate Open Framework) nanocomposite with ~4 V of operation has been developed by a cost effective room temperature synthesis that eliminates any expensive post-synthetic treatments at high temperature under Ar/Ar-H_2_. Firstly, an hydrated nanocomposite, rGO/K_2_[(VO)_2_(HPO_4_)_2_(C_2_O_4_)]·4.5H_2_O has been prepared by simple magnetic stirring at room temperature which releases water to form the anhydrous cathode material while drying at 90 °C during routine electrode fabrication procedure. The pristine MOPOF material undergoes highly reversible lithium storage, however with capacity fading. Enhanced lithium cycling has been witnessed with rGO/MOPOF nanocomposite which exhibits minimal capacity fading thanks to increased electronic conductivity and enhanced Li diffusivity.

Electric vehicles (EVs) are considered as the most environmental-friendly means of transportation for the foreseeable future since they can circumvent the emission of greenhouse gases from burning of fossil fuels in internal combustion engines. In order to realize the large scale production of EVs and to carry out smooth and swift transition from gasoline, the electric vehicles have to overcome serious challenges which include improvement in performance, operational safety and first of all cost reduction. The high price of EVs is mainly due to the high cost of current LIBs, and hence it is mandatory to develop lower cost LIBs with high energy density/power performance and good operational safety^1^,^2^. As the cathode materials account for more than 40% of the total cost of LIBs, the cost reduction should primarily focus on alternative low cost cathode materials^3^.

Various layered oxide-based cathode materials such as LiCoO_2_^4^, Li(Ni,Mn,Co)O_2_^5^,^6^, Li(Ni,Co,Al)O_2_^7^,^8^, and spinel oxide cathodes like Li(Ni,Mn)_2_O_4_^9^,^10^ were developed in the past few decades to power electronic gadgets. Although the safety issues with LiCoO_2_ due to oxygen liberation were mitigated by partial substitution of Co with other metals^5^,^6^,^7^,^8^, olivine-type phosphate framework cathode, LiFePO_4_ developed by Padhi *et al.* in 1997 stood out as the preferred choice for electric vehicle applications owing to its excellent structural stability upon cycling and the cost advantage of Fe over Co^11^. While LiFePO_4_ has a slightly lower capacity of 170 mAh g^−1^ when compared to the layered oxides, it features higher power density, longer cycle life and is more environmental friendly. Despite the inferior electronic and ionic conductivities of pristine LiFePO_4_^12^,^13^, huge improvements were made possible by combined strategies of nanoparticle synthesis with optimum morphology and carbon coating^14^,^15^,^16^. However, its low operating voltage (3.4 V) limits the practical energy density. Extensive research has been carried out to introduce new materials with high power/energy density and to enhance the individual performance of known cathode materials. However, the materials proposed so far require either high temperature synthesis or solvothermal/ microwave conditions followed by expensive post-synthetic calcination at high temperatures, sometimes under Ar or Ar-H_2_ atmosphere for better electrochemical process^17^,^18^,^19^. In addition, while hydrothermal syntheses using autoclaves and microwaves can be optimized for large scale synthesis, they still limit the scaling-up of reactions for commercial use. Hence, it is crucial to find alternative synthetic methods which eliminate the need for costly and difficult-to-scale-up processing steps such as high temperature reactions and post-synthetic calcinations in order to produce low cost electrode materials without compromising on electrochemical performance.

In this work, we report the enhanced electrochemical performance of a metal-organic phosphate open framework (MOPOF) cathode material, rGO/K_2_[(VO)_2_(HPO_4_)_2_(C_2_O_4_)] which has been synthesized at room temperature by magnetic stirring followed by drying at 90^o^C. The oxalatophosphate frameworks, A_2_[(VO)_2_(HPO_4_)_2_(C_2_O_4_)]; (A = Li, Na or K) investigated in our previous studies have transition metal phosphates cross-linked by oxalate ligand^20^,^21^. While many organic materials are considered for reversible lithium storage^22^,^23^,^24^,^25^, these MOPOF materials which are made of organic-inorganic hybrid frameworks can host different alkali cations (Li^+^, Na^+^ and K^+^) between anionic layers and hence used for reversible lithium storage^20^,^21^,^26^,^27^,^28^,^29^. Though these MOPOFs do not require post synthetic high temperature calcination, synthesis under hydrothermal conditions hinders the purpose of low cost production^20^,^21^. In this context, we have explored easier synthetic procedures at low cost. In addition, better electrochemical properties have been also achieved by embedding the particles in reduced graphene oxide (rGO) sheets. As rGO is known to have dual role of downsizing and enhancing the conductivity, the rGO/MOPOF composite has been prepared. In the process of reducing the cost of cathode material production, tartaric acid has been serendipitously found its utility in the synthesis of rGO/K_2_[(VO)_2_(HPO_4_)_2_(C_2_O_4_)] instead of the relatively expensive oxalic acid. The rGO composite exhibits good reversible lithium storage with minimal capacity fading compared to the pristine sample and a capacity of 100 mAh g^−1^ and 57 (±3) mAh g^−1^ with minimal capacity fading was observed at 0.2 and 4 C current rates respectively. Galvanostatic intermittent titration technique (GITT) also revealed the good Li ion diffusion coefficients in the framework, which are of the order of common layered oxide cathodes.

## Results

The MOPOF material, K_2_[(VO)_2_(HPO_4_)_2_(C_2_O_4_)]∙4.5H_2_O was prepared at room temperature by three different techniques, namely, magnetic stirring, mechanical grinding and sonochemical reaction from a 1 : 1.4 : 4 : 20 mixture of V_2_O_5_, tartaric acid, KOH and H_3_PO_4_. Powder X-ray diffraction (PXRD) patterns of the bluish green solid obtained from the three different reactions are shown in Supplementary Fig. 1. Formation of pure phase of K_2_[(VO)_2_(HPO_4_)_2_(C_2_O_4_)]∙4.5H_2_O was evidenced from the exact match of these PXRD patterns with its simulated powder pattern^20^ obtained from single crystal data. In our actual attempts to synthesize a hybrid inorganic-organic framework material containing tartrate and phosphate ligands, tartrate was found to decompose to oxalate resulting in the formation of oxalatophosphate framework as confirmed by X-ray diffraction studies. It is anticipated that V_2_O_5_ catalyses the *in situ* transformation of tartrate to oxalate leading to the precipitation of K_2_[(VO)_2_(HPO_4_)_2_(C_2_O_4_)]∙4.5H_2_O at room temperature. The *in situ* conversion of tartrate^30^ and various other carboxylates (pyridinedicarboxylate^31^,^32^, croconate^33^, orotate^34^ and acetate^35^) to oxalate has been reported in literature which supports this observation. Therefore, the relatively inexpensive tartaric acid was used as an organic source instead of oxalic acid in the systematic synthesis and electrochemical testing of the MOPOF cathode material, K_2_[(VO)_2_(HPO_4_)_2_(C_2_O_4_)].

**Figure 1:**
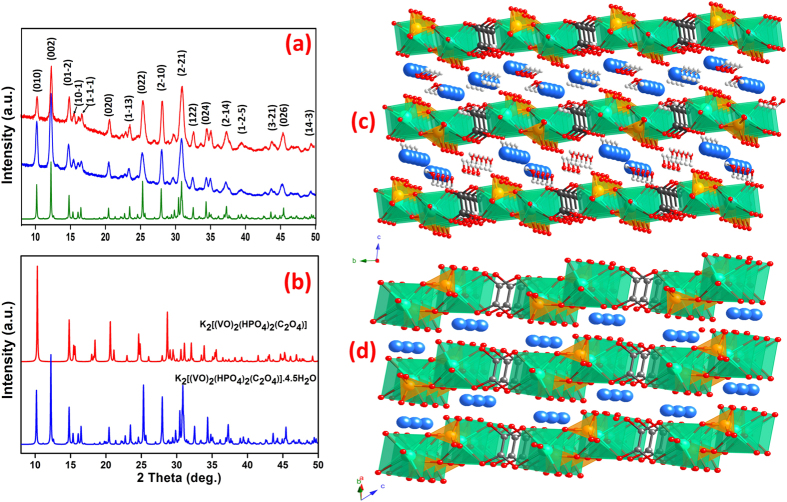
Structure analysis of MOPOF materials. (**a**) Comparison of PXRD patterns of K_2_[(VO)_2_(HPO_4_)_2_(C_2_O_4_)]∙4.5H_2_O (blue) and its rGO nanocomposite (red) with simulated powder pattern from single crystal data (green); (**b**) PXRD patterns of the hydrated phase (blue) and the anhydrous phase (red). Perspective views of the crystal structure of (**c**) hydrated phase, K_2_[(VO)_2_(HPO_4_)_2_(C_2_O_4_)]∙4.5H_2_O and (**d**) anhydrous phase, K_2_[(VO)_2_(HPO_4_)_2_(C_2_O_4_)] showing their layered structure with K^+^ ions in the interlayer space (V, P, K, O, C and H atoms are shown as bluish green, orange, blue, red, black and while balls respectively).

Preliminary electrochemical studies of K_2_[(VO)_2_(HPO_4_)_2_(C_2_O_4_)] prepared by the above-mentioned methods revealed that the sample obtained by magnetic stirring exhibits better lithium storage properties than those produced by sonochemical reaction or mechanical grinding and will be discussed in detail in the electrochemical studies below. Hence, the magnetic stirring methodology was adopted for the synthesis of the rGO composites. The presence of rGO along with the MOPOF material is expected to enhance its electronic conductivity and Li diffusivity and hence to improve the lithium storage capability. The rGO/MOPOF nanocomposite was prepared in a similar way as the pristine sample, with the exception of graphene oxide (GO) addition to the reaction mixture which gets reduced *in situ* during the synthesis. PXRD patterns of the pristine and rGO composite of K_2_[(VO)_2_(HPO_4_)_2_(C_2_O_4_)]∙4.5H_2_O are compared in Fig. 1a. The PXRD patterns matched well with the simulated pattern indicating the formation of phase-pure rGO nanocomposite. The amount of rGO in the samples prepared with lower and higher concentrations of GO were found to be 4 and 8 wt%, respectively, from CHNS analysis.

As the anhydrous phase is used as the cathode material, it is essential to investigate the dehydration behaviour and crystal structure of the anhydrous phase. Supplementary Fig. 2 shows the *in situ* PXRD patterns of the hydrated phase recorded in air from room temperature to 150 °C. The room temperature pattern corresponds to the hydrated phase, K_2_[(VO)_2_(HPO_4_)_2_(C_2_O_4_)]∙4.5H_2_O and an increase in the temperature leads to loss of water, indicated by the decrease of peak intensity at 2θ = 12.2^o^. When the temperature reaches 50 °C, a new peak starts to appear at 2θ = 13.2^o^ which can be explained as the formation of a dihydrate phase, K_2_[(VO)_2_(HPO_4_)_2_(C_2_O_4_)]∙2H_2_O. This is further supported by the loss of ~2.5 moles of water in the thermogravimetric analysis (TGA) (as shown in Supplementary Fig. 3). At 75–80 °C, the dihydrate phase is present alone as the pure phase. The remaining water is lost at ~90 °C, yielding the anhydrous phase, K_2_[(VO)_2_(HPO_4_)_2_(C_2_O_4_)]. This is evidenced by the disappearance of the peak at 2θ = 13.2^o^ and appearance of a new peak at 2θ = 14.8^o^. The identical XRD patterns from 90 to 150 °C indicate that the dehydration process is completed below 90 °C. The PXRD patterns of the hydrated and anhydrous phases are shown in Fig. 1b.

To illustrate the lithium storage mechanism in K_2_[(VO)_2_(HPO_4_)_2_(C_2_O_4_)], it is essential to elucidate its crystal structure. Therefore, the structure of the anhydrous phase was solved and refined from the PXRD pattern collected *in situ* at 120 °C, where the water molecules are removed from the framework completely (More details in the supporting information). Fig. 1c,d show the crystal structures of the hydrated and anhydrous phases respectively. The hydrated phase has a triclinic crystal structure (space group: Pī) with lattice parameters, *a* = 6.3953(4), *b* = 9.1451(5), *c* = 14.6208(9), *α* = 97.269(1), *β* = 91.351(1) and *γ* = 106.500(1). It is characterized by a layered structure made of anionic layers of [(VO)_2_(HPO_4_)_2_(C_2_O_4_)]^2−^ units, which are aligned along the *ab-*plane. The vanadium atoms have a distorted VO_6_ octahedral coordination geometry. The VO_6_ octahedra share corners with three different HPO_4_ to form infinite chains of VOHPO_4_ which in-turn are bridged by C_2_O_4_ ligands in bis-bidendate fashion to form the anionic layers, [(VO)_2_(HPO_4_)_2_(C_2_O_4_)]. These layers are stacked along the *c*-axis which host the K^+^ ions and water molecules between the layers.

Though the anhydrous phase, K_2_[(VO)_2_(HPO_4_)_2_(C_2_O_4_)] is built of the same anionic layers with similar coordination geometries, the packing of the layers is slightly different from that of the hydrated phase. The anhydrous phase also crystallizes in the triclinic space group Pī with the following lattice parameters; *a* = 6.3186, *b* = 7.4869, *c* = 10.8610, *α* = 56.780, *β* = 58.039 and *γ* = 54.204. The difference in all lattice parameters, most pronounced in the direction perpendicular to the anion layers, reflects the loss of 4.5 molecules of water. An extended packing diagram of the crystal structure is shown in Supplementary Fig. 4. The vanadium cation is coordinated to three oxygen atoms from the HPO_4_ moiety, two oxygen atoms from the organic linker C_2_O_4_ and one oxygen atom that is further bonded to the potassium cation (the V–O length to this oxygen atom, 1.68(1) Å, is significantly shorter than the rest of the V–O bonds, 1.80(1) Å–2.28(1) Å, indicating the presence of a VO moiety). The resulting polyhedron can be considered as a distorted octahedron. Each potassium cation is coordinated by seven oxygen atoms (three from the hydrogen phosphate, two from the organic linker and two from the VO moiety). Two symmetry-related potassium cations form edge-shared double polyhedra, which have heavily distorted geometry (Supplementary Fig. 5). Severely distorted polyhedra around potassium and cesium cations are known in the literature^36^. The over-all crystal packing presents a porous metal-organic inorganic hybrid framework, where layers of edge and corner shared polyhedra are connected by the organic linker. The crystal voids are situated around open sites of the potassium cations, enabling ion mobility and migration. The crystal structure was validated by Rietveld refinement (Supplementary Fig. 6). Various crystallographic and Rietveld refinement details of K_2_[(VO)_2_(C_2_O_4_)(HPO_4_)_2_] are listed in Table 1.

**Figure 2:**
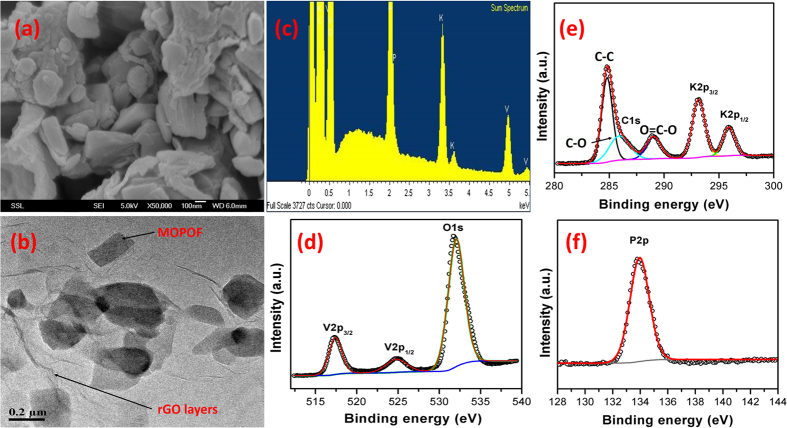
Morphology and composition. (**a**) FESEM image of rGO/K_2_[(VO)_2_(HPO_4_)_2_(C_2_O_4_)]∙4.5H_2_O composite (scale: 100 nm, mag: 50000), (**b**) TEM image of the composite showing the MOPOF nanoplates embedded in rGO layers (scale: 200 nm); (**c**) EDX spectra of K_2_[(VO)_2_(HPO_4_)_2_(C_2_O_4_)]∙4.5H_2_O and (**d**–**f**) XPS spectra of individual elements in K_2_[(VO)_2_(HPO_4_)_2_(C_2_O_4_)]∙4.5H_2_O; (**d**) O1s, V2p; (**e**) K2p, C1s and (**f**) P2p.

Morphology and crystallite size of the prepared materials were investigated by scanning electron microscopy (SEM) and transmission electron microscopy (TEM). The pristine MOPOF sample has particles with plate-like morphology due to its layered structure and the particle size was found to be few microns as evidenced by SEM studies (Supplementary Fig. 7a). The addition of GO in the reaction mixture results in the formation of an rGO/MOPOF nanocomposite with particle size of ~100–200 nm (Fig. 2a). As the aqueous solution of graphene oxide can be mixed uniformly with the aqueous reaction mixture, it resulted in a homogeneous nanocomposite. TEM studies (Fig. 2b and Supplementary Fig. 8) confirmed the presence of rGO layers and the MOPOF nano particles are embedded in these layers. The presence of rGO in the composites are also confirmed by Raman studies (Supplementary Fig. 9). As the MOPOF nanoplates are wrapped by large rGO layers, they form aggregates of few microns in size. In addition, the particle size of the composite increases with increased reaction time as confirmed by the SEM image (Supplementary Fig. 7b). The presence of rGO is expected to improve the lithium storage properties primarily by two ways, (i) it increases the electronic conductivity of the sample and (ii) it helps in the decrease of particle size which results in a better Li diffusion kinetics. The porosity and Brunauer-Emmett-Teller (BET) specific surface area of rGO/MOPOF composites were investigated using N_2_ adsorption-desorption isotherms (Supplementary Fig. 10). The two samples containing 4 and 8% of rGO possess BET surface area of 6.7 and 7.9 m^2^ g^−1^ respectively which can be attributed to the presence of rGO layers and nanosized particles. The pore diameter of the two samples were 12.5 and 11.3 nm respectively. EDX spectrum of the sample shown in Fig. 2c indicates the presence of V, P and K in stoichiometric amounts. X-ray photoelectron spectroscopy (XPS) studies confirm the oxidation state of vanadium atoms as IV. XPS spectra of the different elements such as V, K, P, C and O present in rGO/K_2_[(VO)_2_(HPO_4_)_2_(C_2_O_4_)]∙4.5H_2_O are shown in Fig. 2d–f and the corresponding binding energies are provided in the supporting information.

### Electrochemical studies

**Figure 3:**
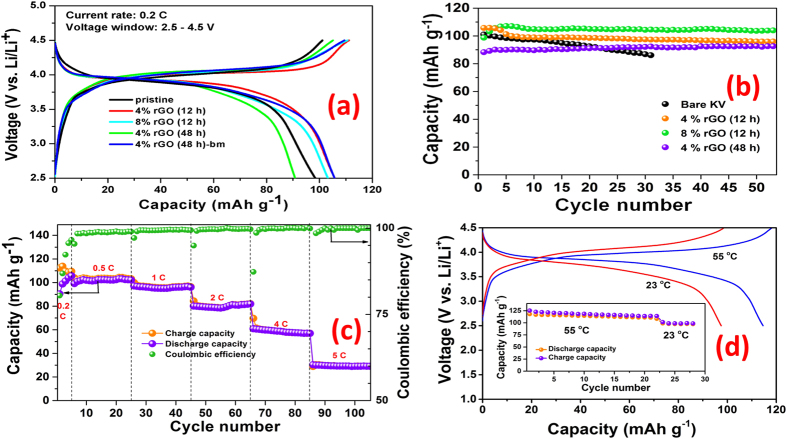
Electrochemical studies. Galvanostatic cycling studies of Li-half cells with rGO/K_2_[(VO)_2_(HPO_4_)_2_(C_2_O_4_)] cathode in the voltage range, 2.5–4.5 V vs. Li/Li^+^; (**a**) voltage *vs.* capacity profiles (2^nd^ cycle) of pristine and different rGO nanocomposites at a current density of 20 mA g^−1^ (~0.2 C current rate); (**b**) Variation of capacity (discharge) with cycle number of the pristine sample and rGO nanocomposites at a current density of 20 mA g^−1^ (~0.2 C current rate); (**c**) Rate capability studies of bm-rGO/K_2_[(VO)_2_(HPO_4_)_2_(C_2_O_4_)] (with 4% rGO) at current rates of 0.2, 0.5, 1, 2, 4 and 5 C where 1 C represents a current density of 108 mA g^−1^ and (**d**) Charge-discharge profiles of bm-rGO/K_2_[(VO)_2_(HPO_4_)_2_(C_2_O_4_)] (with 4% rGO) at high temperature (55 °C) a current density of 20 mA g^−1^ (~0.2 C current rate). The inset figure shows the capacity vs. cycle number plot at 55 °C.

Electrochemical performance of the pristine K_2_[(VO)_2_(HPO_4_)_2_(C_2_O_4_)] and its rGO nanocomposites are shown in Fig. 3a–d. Galvanostatic cycling studies were carried out in the voltage window of 2.5–4.5 V using lithium metal as counter electrode. Preliminary battery testing of the pristine samples prepared by three different techniques, namely, magnetic stirring, mechanical grinding and sonochemical reaction indicated that the sample obtained by magnetic stirring exhibits better lithium storage properties than other two samples (Supplementary Fig. 11). Hence, this methodology has been employed for the synthesis of rGO/K_2_[(VO)_2_(HPO_4_)_2_(C_2_O_4_)] nanocomposites and the material without rGO will be referred as pristine sample.

Charge-discharge profiles (at 0.2 C, 20 mA g^−1^) of the pristine sample and the rGO nanocomposites are shown in Fig. 3a. During the first charge cycle, K^+^ ions are extracted from the framework via a bi-phasic reaction as indicated by a flat plateau at 4.1 V (Supplementary Fig. 12), which results in the bare framework, [(V^V^O)_2_(HPO_4_)_2_(C_2_O_4_)]. The K extraction is accompanied by oxidation of vanadium from V^4+^ to V^5+^. The initial charge capacity is closer to the theoretical capacity, indicating almost complete removal of K from the framework. Upon discharge, Li^+^ ions are preferably inserted into the framework rather than K^+^ ions as they have higher mobility than the latter and was evidenced from *ex situ* EDX studies in our previous report^20^. The first discharge curve exhibits a sloping plateau distinct to the charge curve which indicates a single phase behaviour via the formation of a solid solution, Li_x_[(VO)_2_(HPO_4_)_2_(C_2_O_4_)]. During the second and subsequent cycles, lithium extraction (charge) and lithium re-insertion (discharge) exhibits a similar sloping plateau and shows good reversibility of Li cycling in the framework at 3.9 V.

The pristine sample showed a good initial discharge capacity of ~100 mAh g^−1^ (with respect to the weight of K-containing starting material) and undergoes capacity fading upon cycling as shown in Fig. 3b. The discharge capacity decreases by 13% (87 mAh g^−1^) at the end of just 30 cycles. However, the capacity is still significantly higher than the one reported previously^20^. This enhanced performance may be attributed to the reduced particle size obtained by magnetic stirring while the hydrothermally prepared sample in our previous study contained much larger particles of few hundred microns. Though the decreased particle size helped in improvement of lithium diffusion, electronic conductivity of the sample was poor leading to capacity fading. Hence, rGO nanocomposites of the MOPOF material were prepared to enhance the electronic conductivity which is expected to improve the battery performance. The voltage *vs.* capacity profiles of rGO/K_2_[(VO)_2_(HPO_4_)_2_(C_2_O_4_)] nanocomposites for few selected cycles are shown in Supplementary Fig. 13. Though the charge-discharge curves are similar to those of the pristine MOPOF sample, the rGO composites possess higher capacity retention as seen from Fig. 3b. In addition, specific capacity of the composite with higher rGO content (8%) is better than the sample with 4% rGO. Discharge capacities of 98 and 103 mAh g^−1^ were obtained for the composites with 4 and 8 wt% rGO respectively at the end of 50 cycles. The better performance of the rGO composites can be attributed to the formation of nanoplates embedded in rGO layers and this in turn leads to better electronic and ionic conductivities. In addition, the effect of reaction time on the electrochemical performance was studied. When the reaction time was increased from 12 h to 48 h, the particle size increases as evidenced from Supplementary Fig. 7. This sample showed a discharge capacity of 90 mAh g^−1^ for the fifth cycle which increased to 96 mAh g^−1^ after 50 cycles. The capacity was found to be slightly lower compared to the sample prepared in 12 h. In addition, the sample had poor coulombic efficiency. Therefore, the composite was subjected to low energy ball-milling to just decrease the particle size without destroying the crystal structure. The ball-milled sample provides better performance as seen from Fig. 3a with discharge capacity of 106 mAh g^−1^ and better coulombic efficiency.

Rate capability studies were executed with the ball-milled rGO composite at different current densities of 20, 55, 110, 220, 440 and 550 mA g^−1^ which correspond to current rates of ~0.2, 0.5, 1, 2, 4 and 5 C respectively. The charge-discharge profiles of the sample at these current densities are shown in Supplementary Fig. 14. As the rate increases, polarisation increases as expected. The variation of charge and discharge capacity of the sample with cycle number at the different rates and the corresponding coulombic efficiencies are shown in Fig. 3d. The discharge capacity obtained at these different current rates are 105, 103, 96, 82, 57 and 30 mAh g^−1^ respectively. High coulombic efficiency of >99.5% was also observed for higher current rates.

It is also desirable to have good cycling performance at high temperatures for storage of renewable energy or powering of electric vehicles. Therefore lithium intercalation in the material was investigated at a high temperature of 55 °C. The charge-discharge profiles of the composite at RT and 55 °C are shown in Fig. 3d. The higher capacity of the sample at elevated temperature can be ascribed to the faster Li diffusion kinetics and higher electronic conductivity of the electrode material and higher ionic conductivity of electrolyte with increase in temperature. In addition, the polarisation of the sample decreases at 55 °C. The plot of specific capacity vs cycle number at 55 °C is shown in the inset figure of Fig. 3d. The discharge capacity for the first cycle was 118 mAh g^−1^ which undergo slight fading to 110 mAh g^−1^ after 25 cycles. When the temperature was decreased to 23 °C, the capacity decreases to 98 mAh g^−1^ which indicates the better performance of the material at 55 °C.

**Figure 4:**
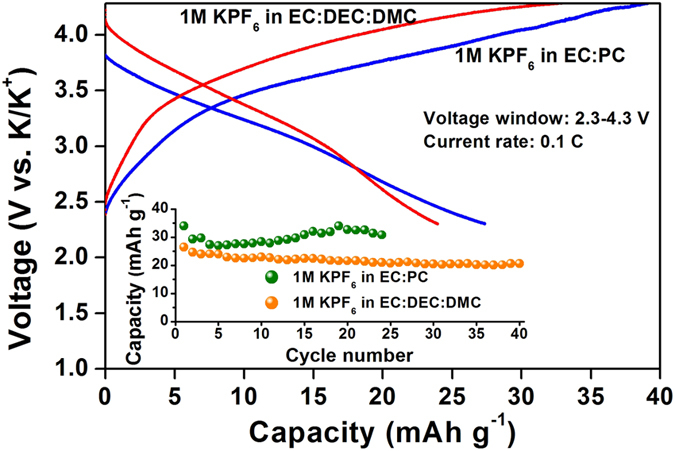
K-intercalation studies of rGO/K_2_[(VO)_2_(HPO_4_)_2_(C_2_O_4_)] composites. Galvanostatic charge discharge cycling showing voltage *vs.* capacity profiles for fifth cycle using different electrolytes in the voltage range, 2.3–4.3 V vs. K/K^+^ at a current density of 11 mA g^−1^ (~0.1 C). The inset figure shows the variation of capacity with cycle number.

The MOPOF material, K_2_[(VO)_2_(HPO_4_)_2_(C_2_O_4_)] originally hosts the K^+^ ions in the layered framework. Therefore, the possibility of reversible K-intercalation in the material was explored by subjecting the rGO/MOPOF cathode to galvanostatic cycling with potassium metal as counter electrode using 1 M KPF_6_ in organic solvents as electrolyte. Figure 4 shows the charge-discharge profiles at a current rate of 0.1 C (11 mA g^−1^). The material exhibits K intercalation by single phase reaction as evidenced from the sloping plateau at an average potential of ~3.4 V which is around 0.6 V lesser compared to the Li cycling. Variation of the specific capacity with cycle number is shown as inset figure in Fig. 4. The reversibility of the K^+^ intercalation in the framework is evident from the figure. When 1 M KPF_6_ in EC, DEC and DMC was used as electrolyte, the initial discharge capacity of 26 mAh g^−1^ was almost retained at 21 mAh g^−1^ after 50 cycles. When 1 M KPF_6_ in EC: PC was used as the electrolyte, it results in slightly better performance. The initial discharge capacity of 34 mAh g^−1^ decreases slightly in the beginning and stabilizes around 32 mAh g^−1^ after 25 cycles as shown in Fig. 4. The larger ionic radius of K^+^ ion is expected to decrease its ionic conductivity which may be explained as the primary reason for the relatively poor K-intercalation in the sample. Careful studies may help to achieve better K intercalation in the material.

## Discussion

Electrochemical lithium storage of the oxalatophosphate framework (MOPOF) cathodes, A_2_[(VO)_2_(HPO_4_)_2_(C_2_O_4_)]; (A = Li, Na or K) were first illustrated in our previous publications^20^,^21^. The reversible lithium storage occurs in framework by de-intercalation of alkali cations (Li, Na or K) from the framework during the first charge cycle from the respective framework while preferable intercalation of Li^+^ ions happens in the first discharge and subsequent cycles as illustrated in the earlier work^20^. Though these MOPOF materials have been proved to undergo reversible Li intercalation at ~4 V due to the reversibility and energetics of V^4+/5+^ redox reaction, the experimental capacities obtained were rather poor (~60% of theoretical capacity). Better capacity and minimal fading were achieved for the rGO/K_2_[(VO)_2_(HPO_4_)_2_(C_2_O_4_)] frameworks in this work due to the enhancement of electronic conductivity of the material due to the presence of the graphene sheets and better lithium diffusivity due to reduced particle size. High reversible capacity of 103 mAh g^−1^ was obtained for the rGO composite with 8 wt% rGO at the end of 50 cycles. Favorably, the materials were also prepared by cost-effective room temperature synthesis which also allows its preparation in large scale compared to the generally used hydrothermal synthesis for the oxalatophosphate compounds.

**Figure 5:**
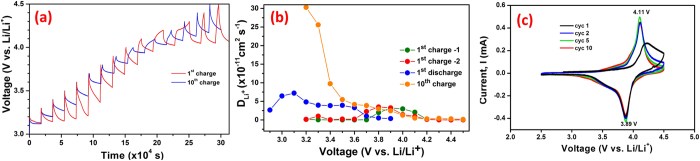
Li-diffusion and CV studies. (**a**) GITT studies of rGO/K_2_[(VO)_2_(HPO_4_)_2_(C_2_O_4_)] cathode; (**b**) Li diffusion coefficient versus cell voltage for the 1^st^ charge and 10^th^ charge cycles and (**c**) CVs of rGO/K_2_[(VO)_2_(HPO_4_)_2_(C_2_O_4_)].

The MOPOF cathode has a layered structure as described earlier which hosts the alkali ions in the interlayer space and allows 2D Li migration. The Li diffusivity is one of the limiting factors in an electrode material to achieve better electrochemical performance. Therefore, the Li-diffusion kinetics in the MOPOF cathode was investigated using galvanostatic intermittent titration technique (GITT). Among the various available methods to study the chemical Li-diffusion behavior of battery materials such as GITT, potentiostatic intermittent titration (PITT), slow-scan rate cyclic voltammetry (SSCV) and electrochemical impedance spectroscopy (EIS), GITT is considered as the most reliable technique. In this method, the electrode material is subjected to lithiation or delithiation by application of a constant current flux for a limited time period τ which results in change of lithium content and the cell voltage increases or decreases to E^o^ depending on the direction of current. Change in the cell voltage during the current flux is calculated by subtracting the IR drop. It is then allowed to relax for a period of 5 h to reach a new steady-state potential (E_s_) and the difference is ΔE_s_. The procedure is repeated as a function of voltage for the entire voltage range, 2.5–4.5 V versus Li/Li^+^. The Li-diffusion coefficient (D_Li_) in the material can be determined from eq. 1 assuming τ << L^2^/D_Li_.


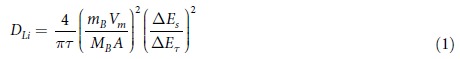


where V_m_ is the molar volume, M_B_ and m_B_ are the molecular weight and mass of active material in the electrode, respectively while A is the geometrical electrolyte-electrode contact area and L is the electrode thickness. The GITT curves for the MOPOF cathode as function of time for the 1^st^ and 10^th^ charge cycles are shown in Fig. 5a. The diffusion coefficients (D_Li_) of the material for the 1^st^ charge, 1^st^ discharge and 10^th^ charge cycles were calculated using eq. 1 and are shown in Fig. 5b as a function of cell potential. The D_Li_ values obtained for the first charge cycle which involves the removal of K^+^ ions from the framework are in the range of 1 × 10^−14^–3 × 10^−12^ cm^2^ s^−1^ for 2 duplicate cells tested in the 2.5–4.5 V potential window. The first discharge cycle in which Li^+^ ions are inserted has D_Li_ in the range, 3 × 10^−12^–7 × 10^−11^ cm^2^ s^−1^ while the 10^th^ charge cycle has D_Li_ in the range of 1 × 10^−12^ to 3 × 10^−10^ cm^2^ s^−1^. The lower diffusion coefficients for the first charge cycle can be attributed to the larger size of K^+^ ions while the subsequent cycle showed higher diffusion coefficients. For comparison, the NASICON type phosphate material, Li_3_V_2_(PO_4_)_3_ GITT studies^37^ find high D_Li_ values of up to 10^−8^ cm^2^ s^−1^ while for the well-studied olivine LiFePO_4_ GITT studies^13^ suggest a low diffusion coefficients of ~10^–15^ cm^2^ s^–1^. The layered oxide cathode materials such as LiCoO_2_ and LiNiO_2_ have D_Li_ values of 10^−8^ to 10^−11^ and 10^−11^ to 10^−12^ cm^2^ s^−1^, respectively^38^,^39^,^40^,^41^. The MOPOF material studied here thus has diffusion coefficients of the similar order to some of the layered oxide cathode materials. Cyclic voltammetry (CV) studies of the rGO/MOPOF cathode are shown in Fig. 5c. The cathodic and anodic peaks of the material are at ~4.1 and 3.9 V respectively, corresponding to the V^4+/5+^ redox reaction. Similar voltage has been observed for the oxalatophosphate and oxalatophosphite systems reported earlier^20^,^21^,^42^. The first cathodic peak is slightly broader at 4.2 V and the different nature is due to the removal of K^+^ ions from the framework while the subsequent cycles involves the insertion/removal of Li^+^ ions. The diffusion coefficients were also determined from CVs of the material using the Randles-Sevcik expression (eq. 2).


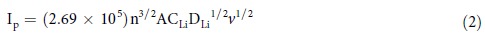


where I_p_ is the peak current in A, n is the number of electrons involved in the redox reaction, A is the area of electrode in cm^2^, D_Li_ is the diffusion coefficient in cm^2^ s^−1^, C is the bulk concentration of the electroactive species in mol cm^−3^ and *v* is the scan rate in V s^−1^. The D_Li_ values obtained from eq. 2 are of the order of 10^−11^ cm^2^ s^−1^ in harmony to the GITT results.

In summary, the MOPOF cathode material, K_2_[(VO)_2_(HPO_4_)_2_(C_2_O_4_)] and its rGO nanocomposite were prepared cost effectively in aqueous solution at room temperature in gram scale followed by dehydration at moderate temperatures below 90 °C. In addition, naturally abundant tartaric acid was used as the organic source which also helps in the synthesis at room temperature itself. The *in situ* decomposition of tartaric acid to oxalate triggers the formation of the oxalatophosphate framework at room temperature. The pristine sample prepared by this method exhibits higher electrochemical activity than the hydrothermally prepared sample in our previous study which can be attributed to the smaller particle size. However, the bare MOPOF material exhibits capacity fading due to its intrinsic poor electronic conductivity. Further improvements in the electrochemical performance of the material has been witnessed through the preparation of rGO/K_2_[(VO)_2_(HPO_4_)_2_(C_2_O_4_)] nanocomposites by the room temperature synthesis. The rGO composites prepared by the simultaneous *in situ* reduction of GO in the reaction mixture leads to the formation of homogenous nanocomposites with nanoplates of the MOPOF material wrapped by the rGO layers. The rGO composite exhibits good reversible lithium storage capacity of 100 mAh g^−1^ and 57 (±3) mAh g^−1^ with minimal capacity fading at 0.2 and 4 C current rates respectively. In addition, the composite possesses good electrochemical activity at high temperature of 55 °C. The MOPOF material studied here has good Li ion diffusion coefficients, which are of similar order of some layered oxide cathodes. This is further supported by the stable performance of the rGO composite after 50 cycles and the reasonably better capacities obtained at higher C rates. These results reinstate that it is highly possible to achieve the maximum extraction/insertion of Li ions from the MOPOF system by reducing the particle size and enhancing the electronic conductivity by embedding in rGO sheets. Though the specific capacity of the material is slightly lower than the inorganic oxide based cathodes, its advantages such as high voltage (4 V) and its cost-effective synthesis at room temperature in aqueous solution can be of high value for utilization in applications where the low-cost is preferred over energy density.

## Methods

### Synthesis of K_2_[(VO)_2_(HPO_4_)_2_(C_2_O_4_)]

The MOPOF cathode material, K_2_[(VO)_2_(HPO_4_)_2_(C_2_O_4_)] was synthesized in this study at room temperature in aqueous solution. The reaction yielded a hydrated phase, K_2_[(VO)_2_(HPO_4_)_2_(C_2_O_4_)]∙4.5H_2_O which undergoes dehydration at 90 °C, leading to the anhydrous cathode material. In a typical synthesis, a brown aqueous solution was formed by magnetic stirring of a 1: 1.4: 4 mixture of vanadium pentoxide (Aldrich, 98%), tartaric acid (Merck, 99.5%) and potassium hydroxide (BDH, 99%) in distilled water. Followed by this, 20 molar ratio of phosphoric acid (85%) was slowly added and stirred continuously for another 12 hours which resulted in a bluish green precipitate of the hydrated phase. In this reaction, the tartrate ligand was found to undergo *in situ* decomposition to oxalate, resulting in the oxalatophosphate framework as shown in eq. 3. The resultant precipitate was filtered, washed with distilled water and dried in air.


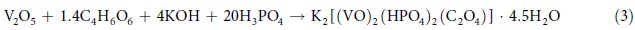


K_2_[(VO)_2_(HPO_4_)_2_(C_2_O_4_)]∙4.5H_2_O was also prepared by mechanical grinding and sonochemical reaction. The reactants in the above mentioned ratio were added to an electric mortar grinder (RM 100, Retsch) and ground for 30 min using minimum quantity of distilled water. A similar bluish green precipitate was obtained which was filtered and washed with distilled water. For the synthesis by sonochemical reaction, an aqueous solution was prepared by sonication of a mixture of V_2_O_5_, tartaric acid and KOH in the molar ratio of 1: 1.4: 4 in distilled water for few minutes in an ultrasonic bath. After a clear solution was obtained, H_3_PO_4_ was added and sonication of the mixture was continued for another 30 minutes which resulted in a bluish green precipitate of the desired product. The anhydrous phase, K_2_[(VO)_2_(HPO_4_)_2_(C_2_O_4_)] was obtained by dehydration of the hydrated phase at 90 °C and used for electrochemical studies.

### Synthesis of rGO/K_2_[(VO)_2_(HPO_4_)_2_(C_2_O_4_)]

Preparation of graphene oxide from graphite powder has been reported earlier^43^,^44^,^45^. Synthesis of the nanocomposite, rGO/K_2_[(VO)_2_(HPO_4_)_2_(C_2_O_4_)]∙4.5H_2_O was carried out at room temperature by a similar magnetic stirring reaction. In a typical synthesis, 50 or 100 mL of GO solution (0.1 wt%) was added to an aqueous solution containing vanadium pentoxide, tartaric acid and potassium hydroxide in a molar ratio of 1 :1.4: 8 and stirred for 2 hours. To the resulting brown solution, phosphoric acid (20 molar ratio) was added and stirred continuously for 12 hours to obtain the greyish precipitate of rGO/[K_2_(VO)_2_(HPO_4_)_2_(C_2_O_4_)]∙4.5H_2_O. Higher quantity of KOH was used in the preparation of rGO composite to adjust the pH to ~3. Elemental analysis determined the rGO content in the two samples as 4 and 8%. The resultant precipitate was filtered, washed with distilled water and dried in air.

#### Materials Characterization

The synthesized compounds were characterized by powder X-ray diffraction (PXRD) using PANalytical or Siemens D5000 diffractometer employing Cu-Kα radiation. For the crystal structure determination, PXRD pattern of K_2_[(VO)_2_(C_2_O_4_)(HPO_4_)_2_] was collected at 120 °C on a Stoe Stadi-P high-resolution laboratory powder diffractometer using primary beam Johann-type Ge(111) monochromator for Cu-*K*α_1_-radiation with the sample placed in a borosilicate capillary (More details in the supporting information). Brunauer-Emmett-Teller (BET) surface area of the rGO composites were determined from N_2_ adsorption-desorption isotherms at 77 K recorded on Tristar 3000 (Micromeritics, USA) analyzer. The samples were preheated for 2 h at 180 °C under nitrogen flow to remove adsorbed moisture prior to BET analysis. Morphology of the samples was examined by scanning electron microscopy (SEM) and Transmission electron Microscopy (TEM). SEM micrographs of the platinum coated samples were recorded using a JEOL JSM-6700F field emission scanning electron microscope (FESEM) operated at 5 kV and 10 μA. JEOL JEM 2010 (operated at 200 kV) was used to record the TEM images of the compounds to determine the surface morphology and particle size. X-ray photoelectron spectra (XPS) of the material were recorded using AXIS ultra DLD spectrometer (Kratos Analytica) with monochromatic Al-Kα radiation.

#### Electrochemical characterization

Electrochemical properties of the samples were investigated using coin cells (type 2016) with Li metal (Kyokuto Metal Co., Japan) as counter electrode, glass microfiber filter (GF/F, Whatman Int. Ltd., Maidstone, England) as the separator and 1 M LiPF_6_ in ethylene carbonate (EC) and diethyl carbonate (DEC) (1:1 v/v, Merck) as the electrolyte. For the potassium intercalation studies, potassium metal cut from lumps were used as the counter electrode. 1 M KPF_6_ in EC and PC (1:1 v/v) or 1 M KPF_6_ in EC, DEC and DMC (1:1:1 v/v) were prepared inside a glove box by dissolving the required amounts of KPF_6_ in the respective organic solvents and were used as electrolytes.

Firstly, a slurry was prepared by mixing the MOPOF sample (70 wt%) with super P carbon black (15 wt%) and 15 wt% of PVDF binder (Kynar 2801) in N-methyl pyrrolidinone (NMP) solvent. The slurry was then coated onto an etched aluminium foil, dried at 90 °C and cut into circular discs of 16 mm diameter. Coin cells were assembled in an Ar-filled glove box (MBraun, Germany) with oxygen and water concentration maintained below 1 ppm, by crimp sealing the thus fabricated cathode with lithium and potassium metal as counter electrode for Li cycling and K cycling respectively. The cells were aged for 12 h before they were subjected to the electrochemical testing. Cyclic voltammetry and galvanostatic discharge−charge cycling studies of the cells were carried out using computer controlled MacPile II (Bio-logic, France) and Bitrode multiple battery tester (model SCN, Bitrode, U.S.A.), respectively.

## Additional Information

**How to cite this article**: Shahul Hameed, A. *et al.* Room temperature large-scale synthesis of layered frameworks as low-cost 4 V cathode materials for lithium ion batteries. *Sci. Rep.* **5**, 16270; doi: 10.1038/srep16270 (2015).

## Supplementary Material

Supplementary Information

## Figures and Tables

**Table 1:** Selected crystallographic and Rietveld refinement details of K_2_[(VO)_2_(C_2_O_4_)(HPO_4_)_2_].

Compound name	DipotassiumBis(vanadylhydrogenphosphate) Oxalate
Molecular formula	K_2_[(VO)_2_(C_2_O_4_)(HPO_4_)_2_]
Sum formula	K_2_V_2_P_2_O_14_C_2_H_2_
Formula weight (g mol^−1^)	492.05
Crystal system	Triclinic
Space group	*P*ī (2)
*Z*	2
*a*/Å	6.316(5)
*b*/Å	7.489(9)
*c*/Å	10.861(6)
α/°	56.8(1)
β/°	57.99(7)
γ/°	54.20(3)
*V*/Å^3^	331.7(6)
Temperature (°C)	120
Wavelength (Å)	1.54059
*R*-exp (%)	1.06
*R*-p (%)	1.96
*R*-wp (%)	2.56
*R*-Bragg (%)	0.56
Starting angle (° 2*θ*)	6.0
Final angle (° 2*θ*)	70
Step width (° 2*θ*)	0.015
Time/scan (hrs)	24
No. of variables	59
