# Supplementary material for: Room temperature large-scale synthesis of layered frameworks as low-cost 4 V cathode materials for lithium ion batteries
Source: Sci Rep. 2015 Nov 23;5:16270. doi: 10.1038/srep16270 (PMC4655412; doi:10.1038/srep16270)
Supplement: Supplementary Information [file srep16270-s1.doc]

**Supplementary Information**

**Room temperature large-scale synthesis of layered frameworks as low-cost 4 V cathode materials for lithium ion batteries**

A. Shahul Hameed,^a^ M. V. Reddy,*^b,c^ M. Nagarathinam,^a^ Tomče Runčevski,^d^ Robert E Dinnebier,*^d^ Stefan Adams,^c^ B. V. R. Chowdari^b^ and Jagadese J. Vittal *^a^

*^a^Department of Chemistry, 3 Science drive 3, National University of Singapore, Singapore 117543.*

*^b^Advanced batteries lab, Department of Physics, 2 Science drive 3, National University of Singapore, Singapore 117551.*

*^c^Department of Materials Science and Engineering, 9 Engineering drive 1, National University of Singapore, Singapore 117575.*

*^d^Max Planck Institute for Solid State Research, Heisenbergstrasse 1, 70569 Stuttgart, Germany*

*Corresponding authors

Email addresses: [chmjjv@nus.edu.sg](mailto:chmjjv@nus.edu.sg), [phymvvr@nus.edu.sg](mailto:phymvvr@nus.edu.sg), [r.dinnebier@fkf.mpg.de](mailto:r.dinnebier@fkf.mpg.de).


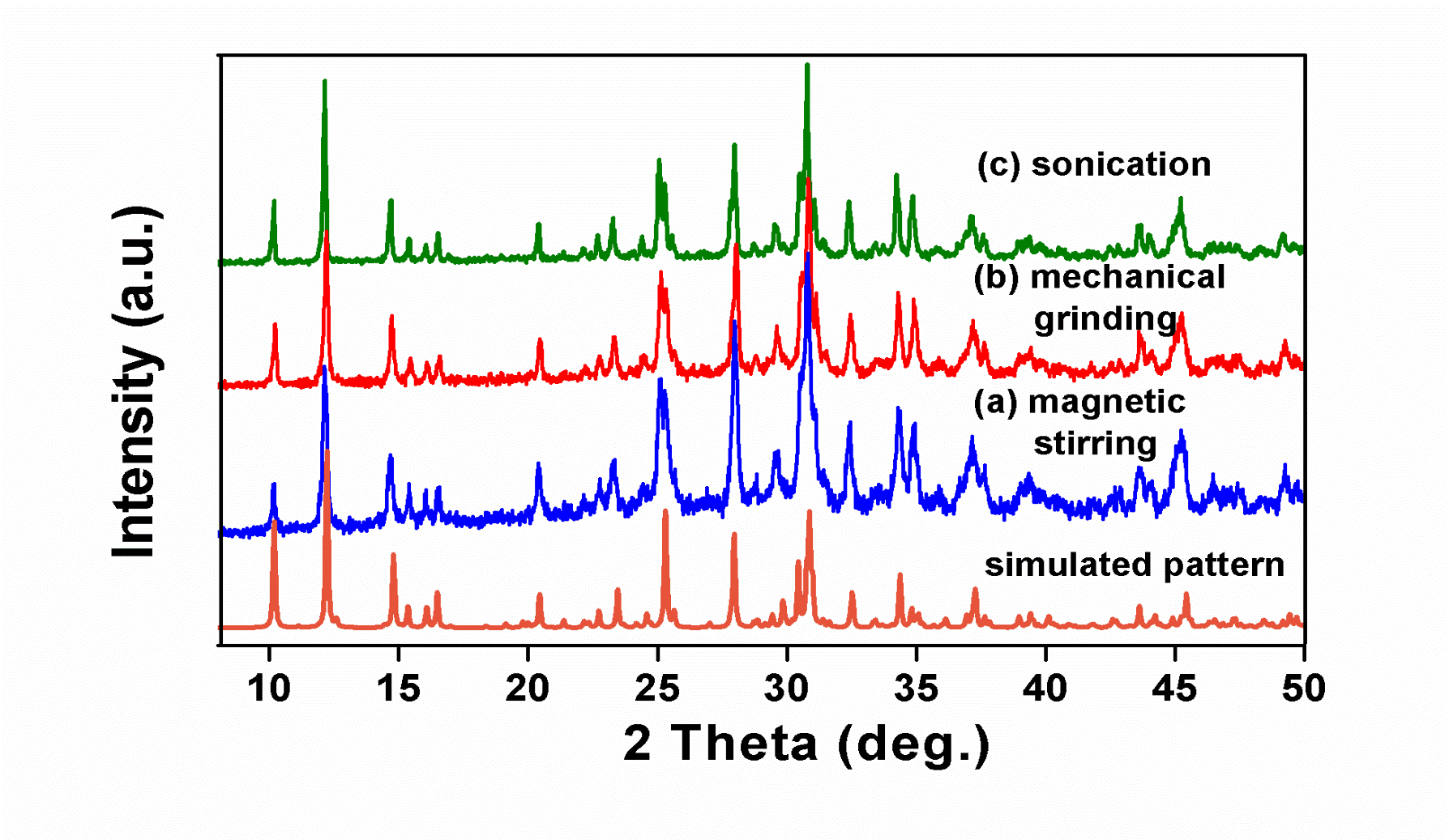


**Supplementary Figure 1 |** PXRD patterns of K_2_[(VO)_2_(HPO_4_)_2_(C_2_O_4_)]∙4.5H_2_O prepared by (a) magnetic stirring; (b) mechanical grinding and (c) sonochemical synthesis compared with its simulated pattern.


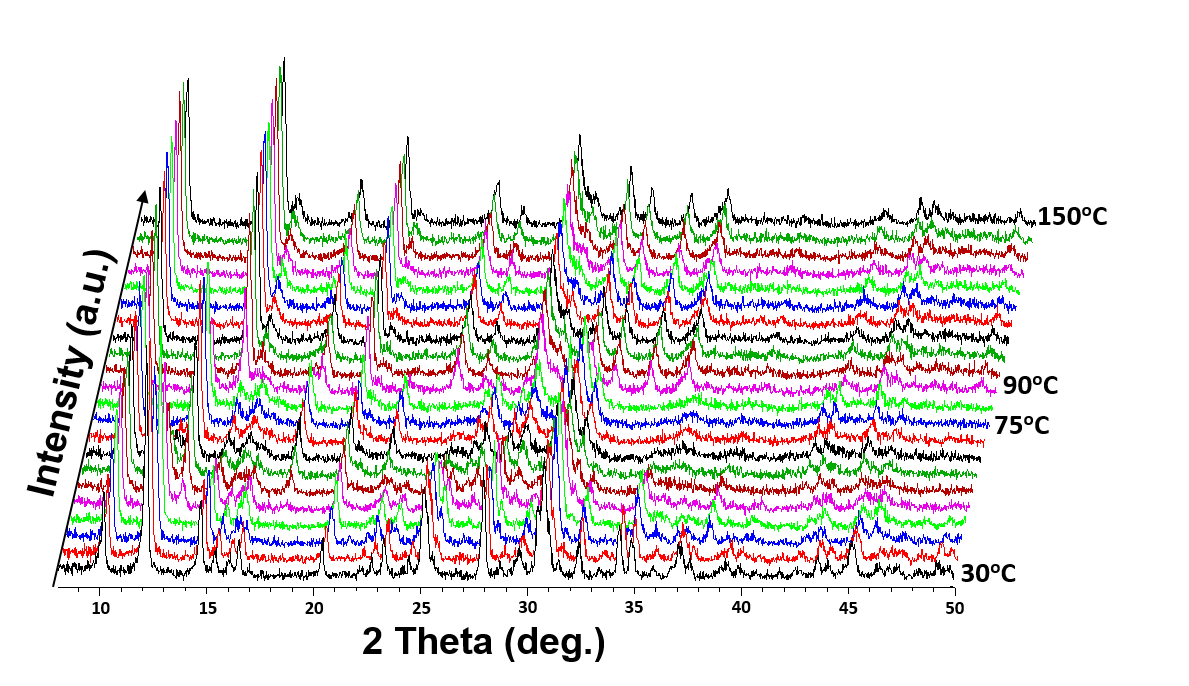


**Supplementary Figure 2 |** In-situ PXRD patterns of K_2_[(VO)_2_(HPO_4_)_2_(C_2_O_4_)]∙4.5H_2_O recorded in air between 30 and 150^o^C.


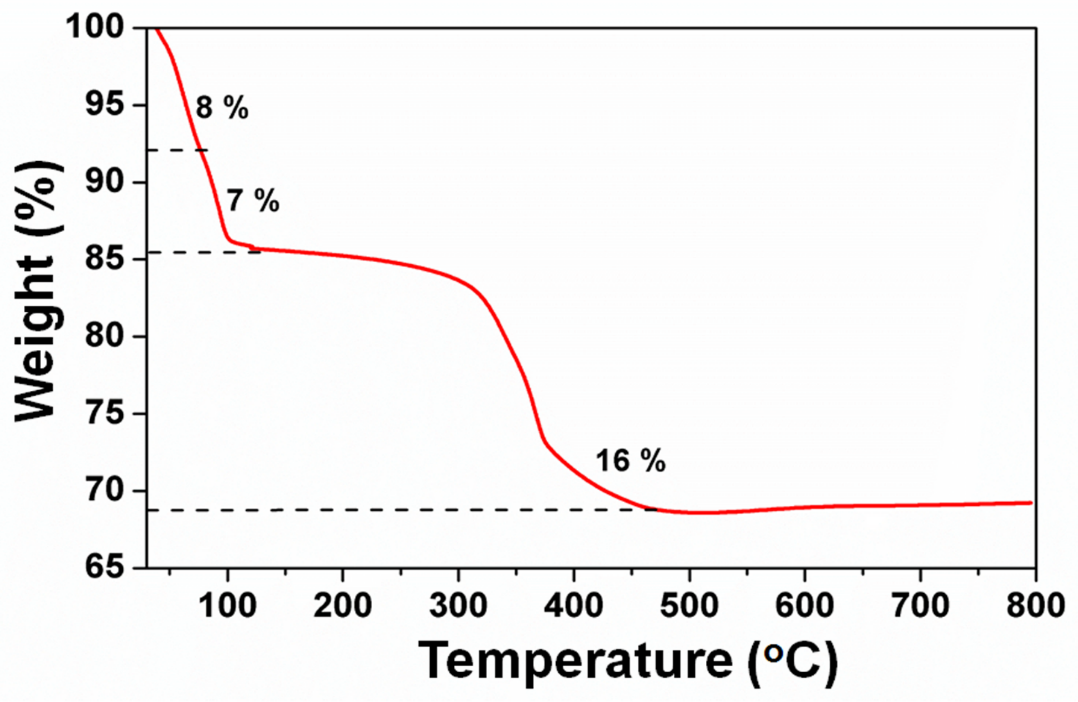


**Supplementary Figure 3 |** TGA of K_2_[(VO)_2_(HPO_4_)_2_(C_2_O_4_)]∙4.5H_2_O in N_2_ flow at a heating rate of 5 ^o^C min^-1^


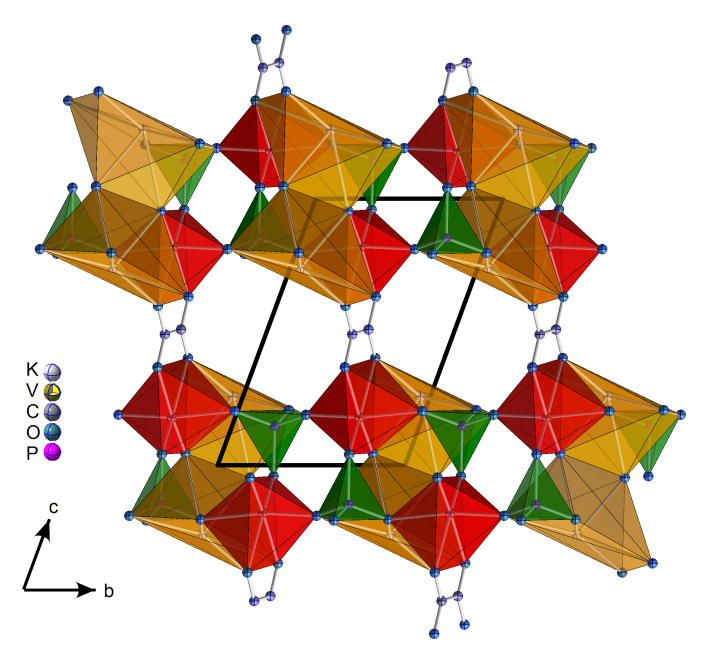


Supplementary Figure 4 | Extended packing motif of K_2_[(VO)_2_(C_2_O_4_)(HPO_4_)_2_]


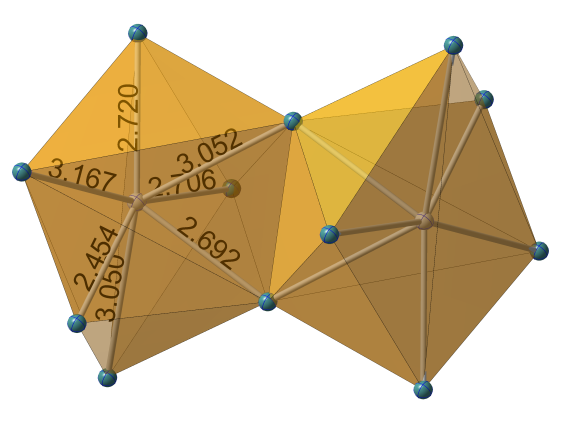


Supplementary Figure 5 | Coordination sphere around the potassium cation in K_2_[(VO)_2_(C_2_O_4_)(HPO_4_)_2_].


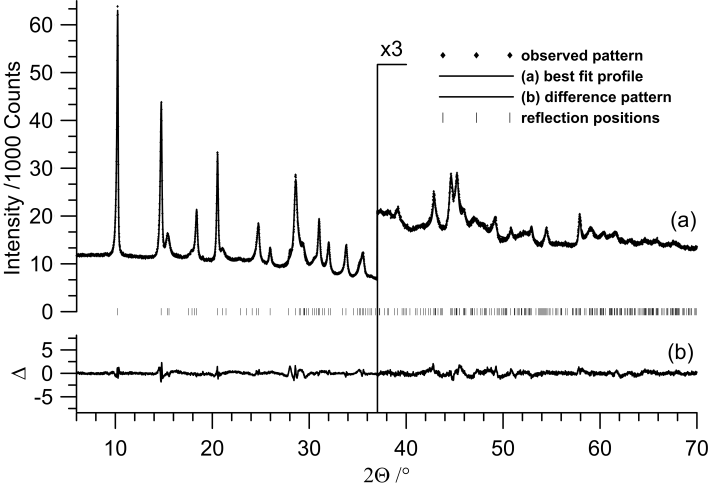


Supplementary Figure 6 | Scattered X-ray intensities of K_2_[(VO)_2_(C_2_O_4_)(HPO_4_)_2_] at 120 °C, presented as a function of diffraction angle. The observed pattern (diamonds) measured in Debye-Scherrer geometry, the best Rietveld fit profiles (line) and the difference curve between the observed and the calculated profiles (below) are shown. The high angle part is enlarged for clarity.


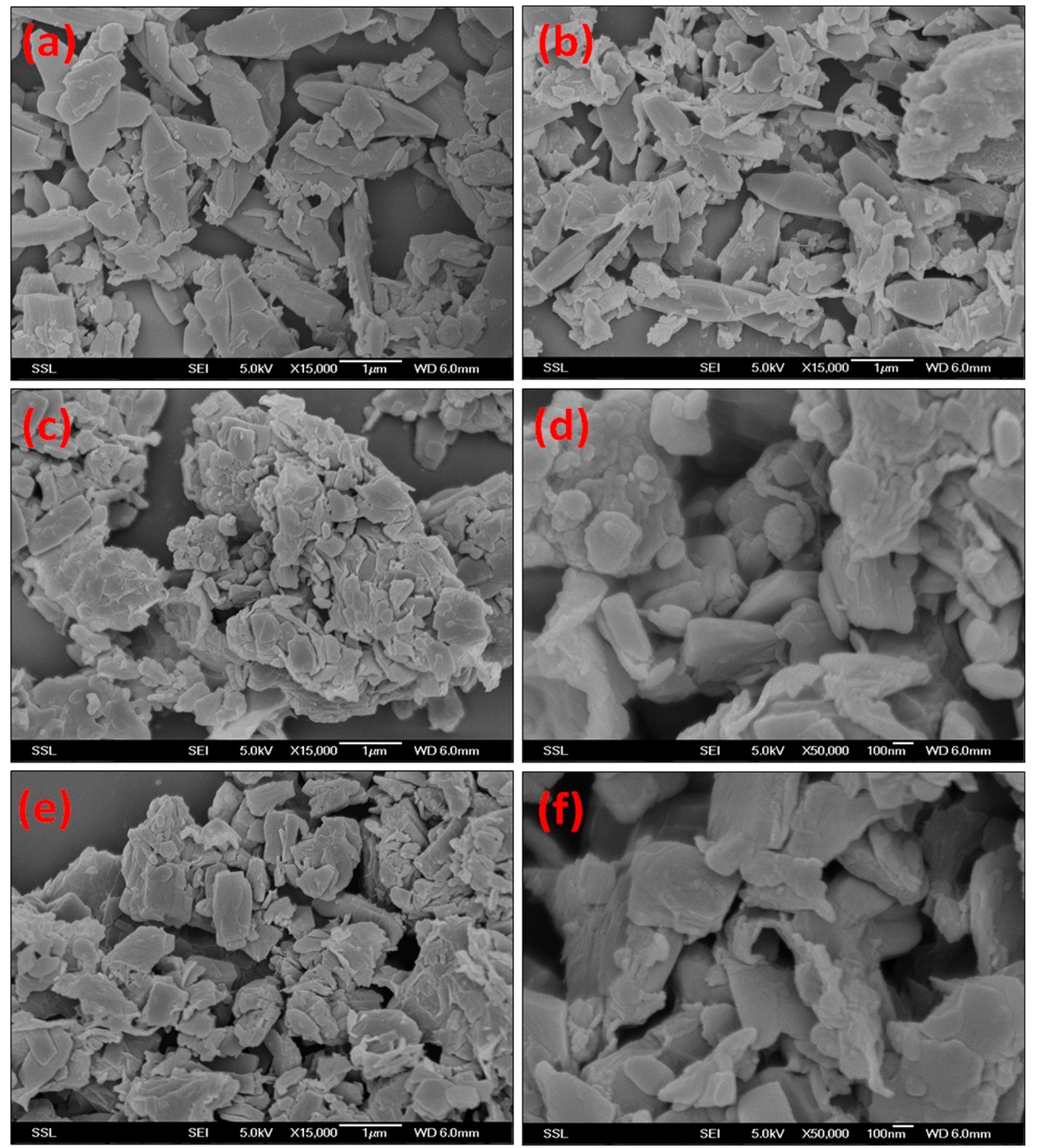


**Supplementary Figure 7 |** SEM images of (a) bare [K_2_(VO)_2_(HPO_4_)_2_(C_2_O_4_)]∙4.5H_2_O; (b) rGO/[K_2_(VO)_2_(HPO_4_)_2_(C_2_O_4_)]∙4.5H_2_O containing 4 % rGO prepared in 48 h; (c & d) rGO composite (4 % rGO) prepared in 12 h and (e & f) rGO composite (8 % rGO) prepared in 12 h


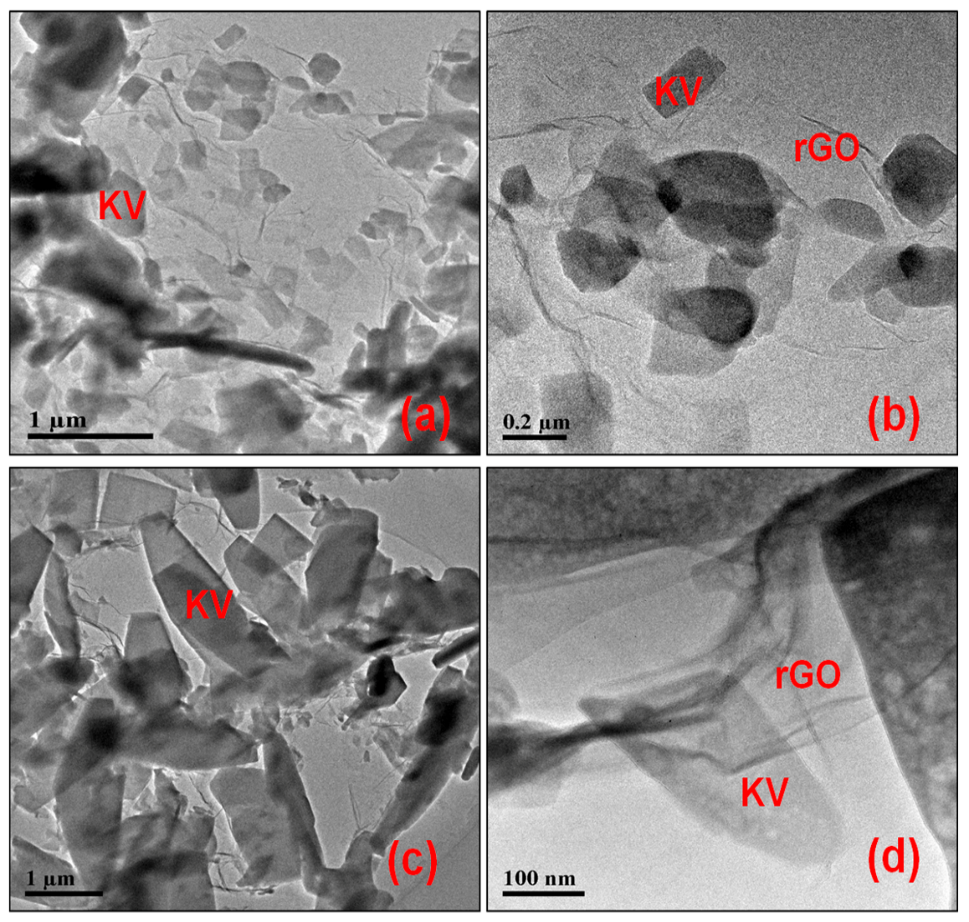


**Supplementary Figure 8 |** TEM images of rGO/[K_2_(VO)_2_(HPO_4_)_2_(C_2_O_4_)]∙4.5H_2_O containing 4 % rGO prepared in 12h (a & b), prepared in 48 h (c & d).


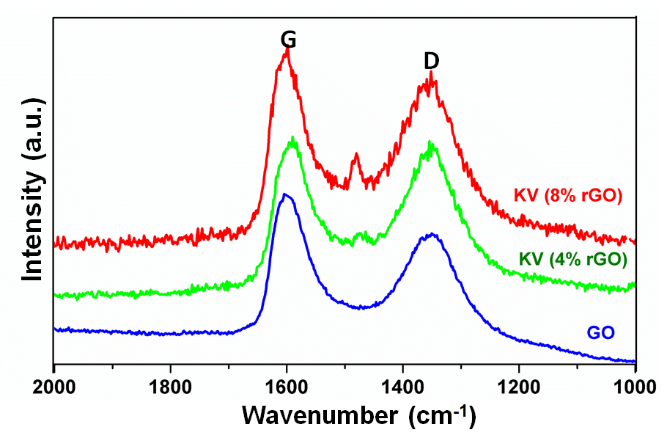


**Supplementary Figure 9 |** Raman spectra of bare GO and rGO/MOPOF composites with 4 and 8 wt % rGO


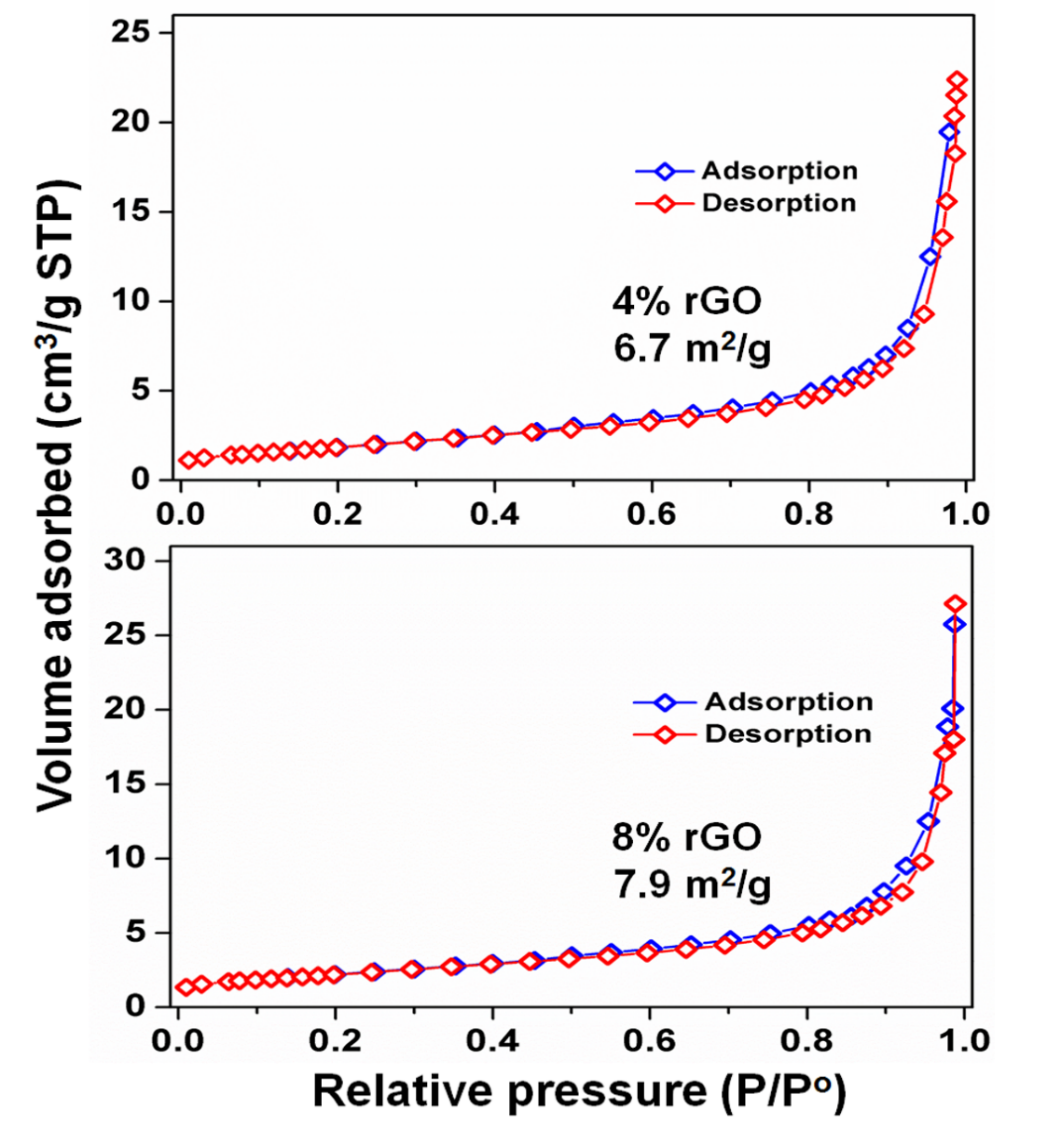


**Supplementary Figure 10 |** Nitrogen adsorption-desorption isotherms of rGO/K_2_[(VO)_2_(HPO_4_)_2_(C_2_O_4_)]∙4.5H_2_O composites containing 4 and 8 % rGO

**
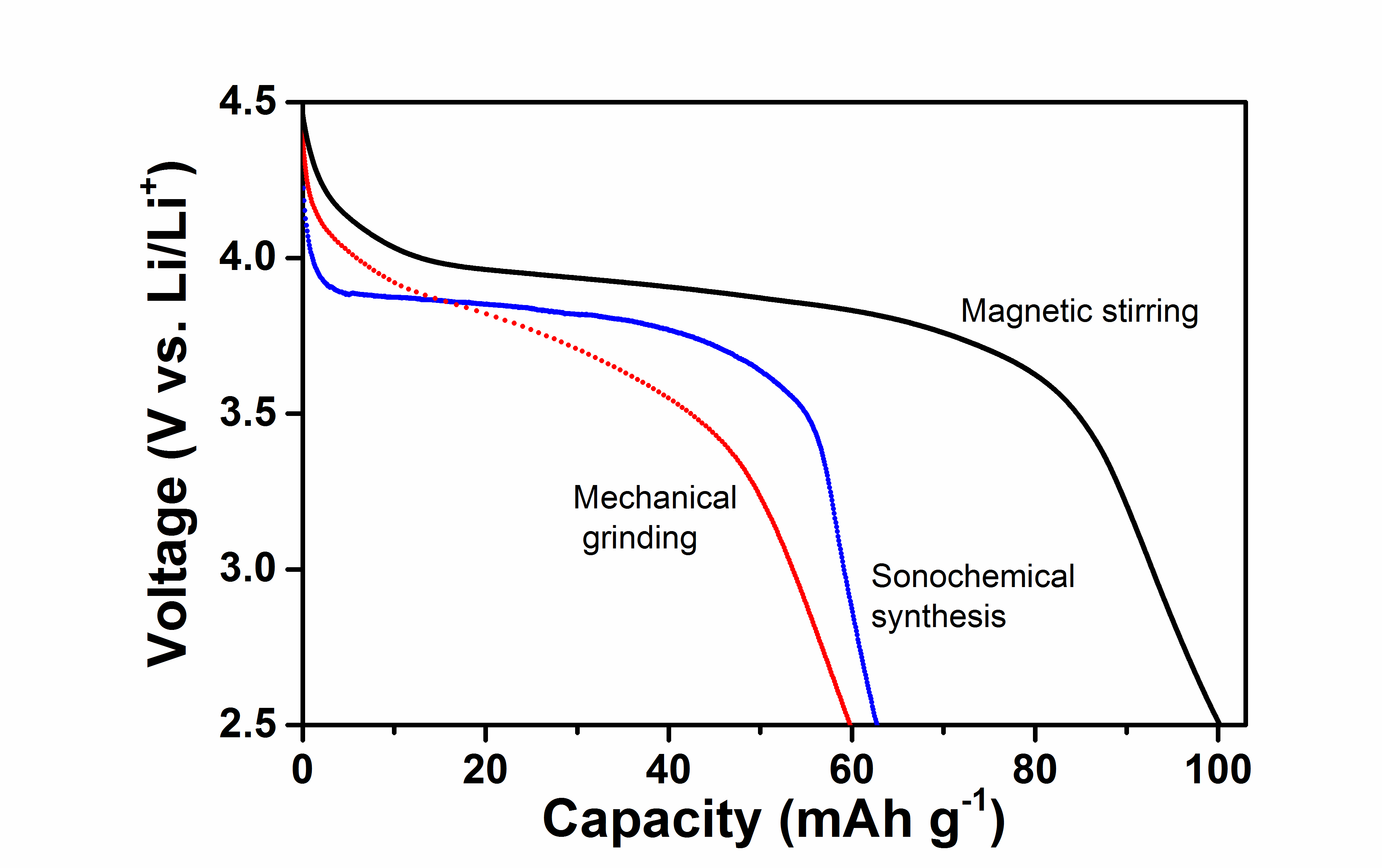
**

**Supplementary Figure 11 |** Galvanostatic cycling studies of pristine K_2_[(VO)_2_(HPO_4_)_2_(C_2_O_4_)] prepared by magnetic stirring, grinding and sonochemical studies in the voltage range, 2.5-4.5 V at a current density of 20 mA g^-1^ (~0.2 C)


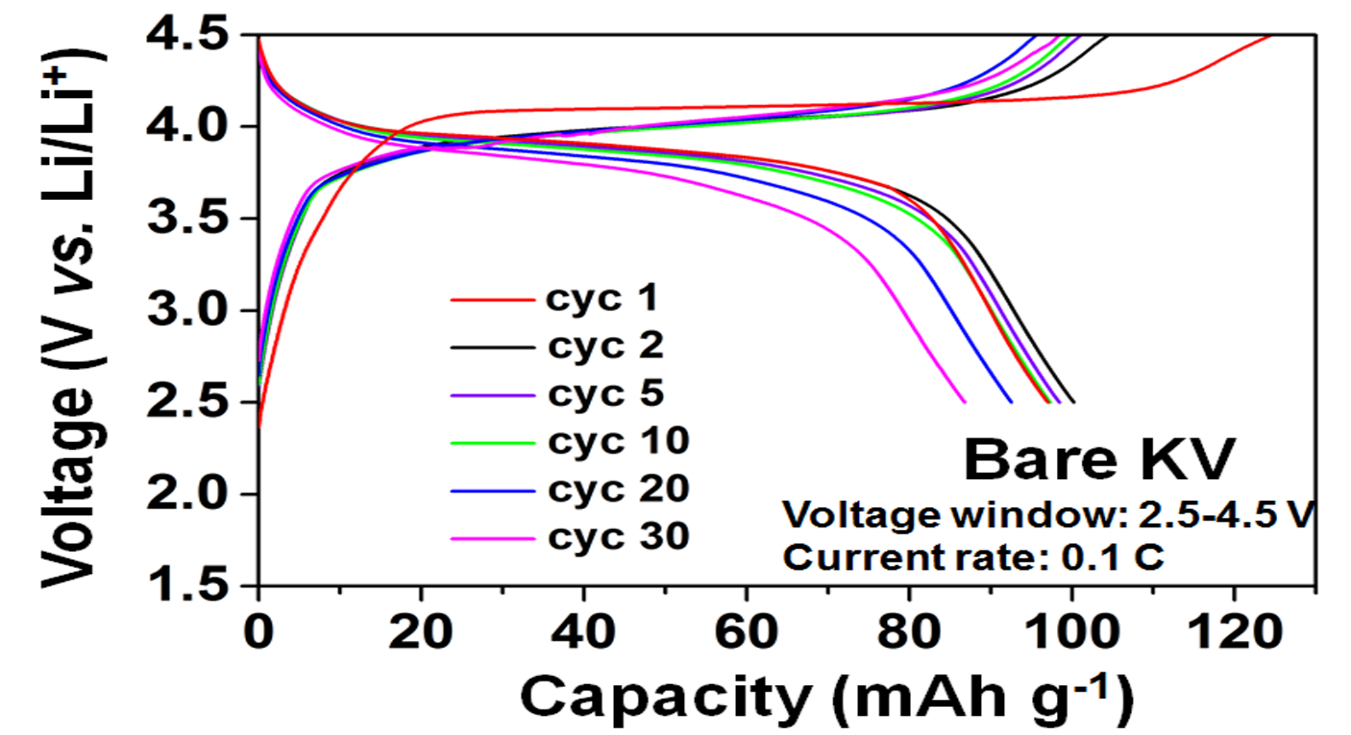


**Supplementary Figure 12 |** Galvanostatic cycling studies of pristine K_2_[(VO)_2_(HPO_4_)_2_(C_2_O_4_)] sample in the voltage range, 2.5-4.5 V at a current density of 20 mA g^-1^ (~0.2 C), showing voltage *vs.* capacity profiles for selected cycles


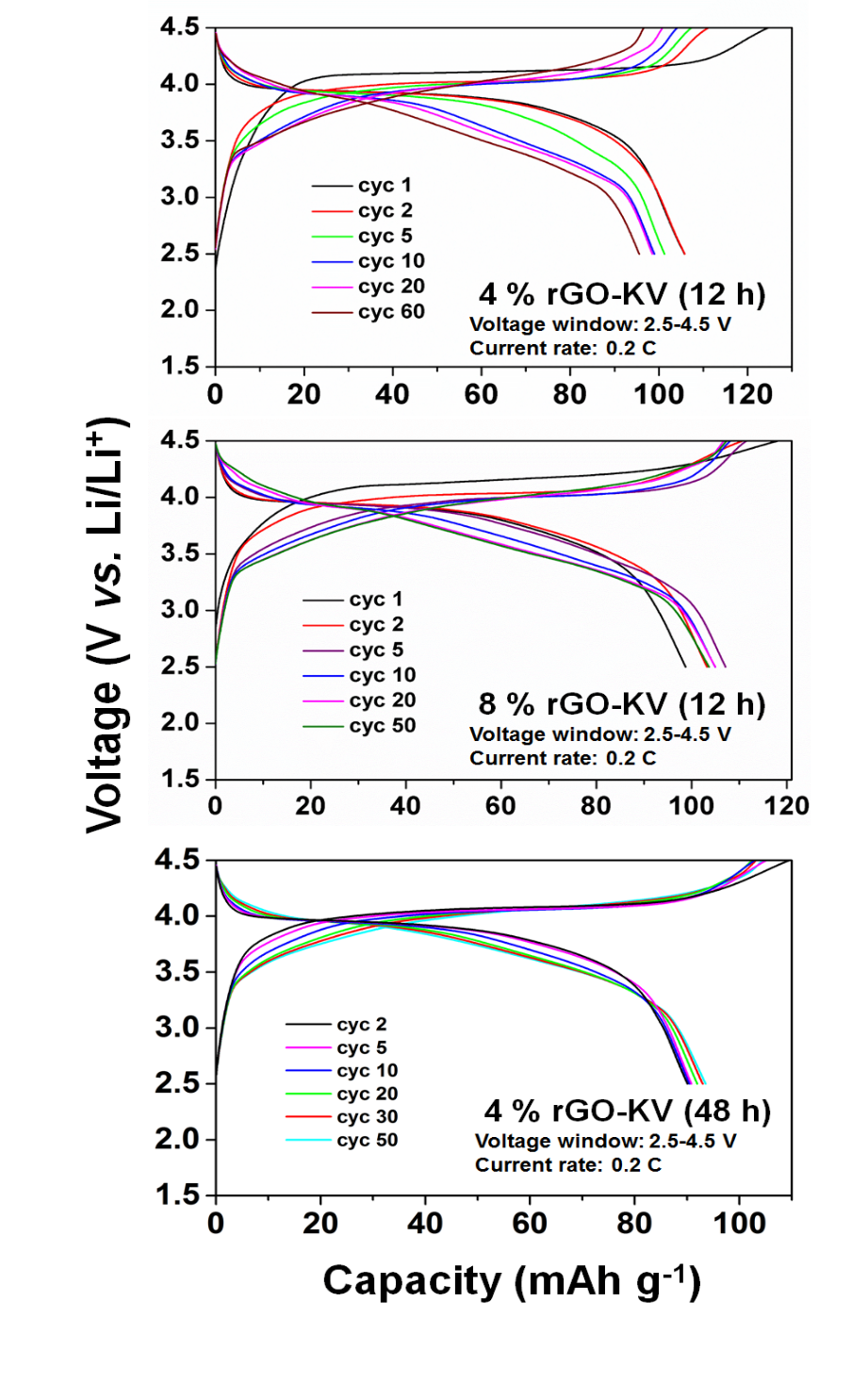


**Supplementary Figure 13 |** Galvanostatic cycling studies of different rGO/K_2_[(VO)_2_(HPO_4_)_2_(C_2_O_4_)] composites in the voltage range, 2.5-4.5 V at a current density of 20 mA g^-1^ (~0.2 C), showing voltage *vs.* capacity profiles for selected cycles.


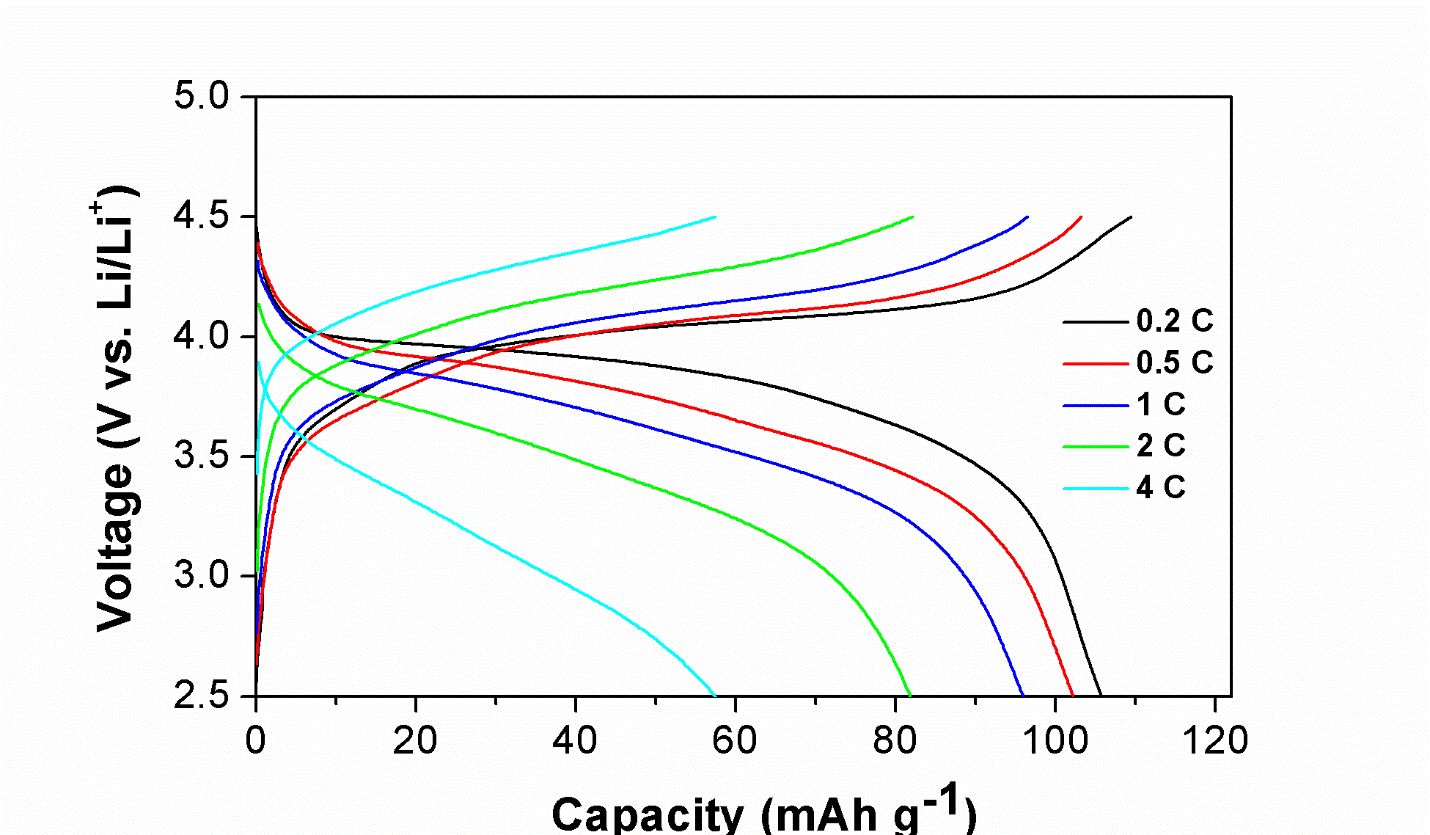


**Supplementary Figure 14 |** Galvanostatic cycling studies of different rGO/ K_2_[(VO)_2_(HPO_4_)_2_(C_2_O_4_)] composites in the voltage range, 2.5-4.5 V at a current density of 20 mA g^-1^ (~0.2 C), showing voltage *vs.* capacity profiles for selected cycles.

**Thermogravimetric analysis (TGA)**

TGA of K_2_[(VO)_2_(HPO_4_)_2_(C_2_O_4_)]∙4.5H_2_O (Fig. S3) was carried out between RT and 900^o^C. It shows two continuous weight loss steps before 100^o^C, which correspond to the removal of 4.5 water molecules from the parent phase. In the first weight loss step, 2.5 H_2_O molecules (8 %) are lost while the second weight loss of ~ 7 % corresponds to the removal of remaining two molecules of water. (Observed, 14.6 %, Calculated, 14.2 %). This can be interpreted as the formation of dihydrate phase and the anhydrous phase during first and second weight loss steps respectively. This is in agreement with the in-situ XRD results (see main article). The weight loss between ~300 and 450 ^o^C can be interpreted as the loss of oxalate ligand (Observed, 16.1 %, Calculated, 15.3 %). From the TGA and in-situ XRD results, the ideal temperature for dehydration of K_2_[(VO)_2_(HPO_4_)_2_(C_2_O_4_)]∙4.5H_2_O was found to be ~ 120^o^C.

Structure determination of K_2_[(VO)_2_(C_2_O_4_)(HPO_4_)_2_]

Powder X-ray Diffraction (PXRD) Data Collection

PXRD pattern of K_2_[(VO)_2_(C_2_O_4_)(HPO_4_)_2_] was collected at temperature of 120°C on a Stoe Stadi-P high-resolution laboratory powder diffractometer in transmission mode (using primary beam Johann-type Ge(111) monochromator for Cu-*K*α_1_-radiation) with the sample placed in a borosilicate capillary. The capillary was spun during data collection for better particle statistics. The powder pattern was recorded for 24 h in the range from 6-70° 2*θ* with a step width of 0.015° 2*θ* using a linear position sensitive silicon strip detector (Mythen-Dectris) with an opening of approximately 12° 2*θ* (further details are given in Table 1, main article). For elevating the temperature to 120°C, a hot-air blower (Oxfords Cryostream 700+) was used.

Crystal Structure Solution and Refinement

For indexing of the powder pattern, as well as for solution and refinement of the crystal structure, the program TOPAS version 4.2 (Bruker-AXS, 2007) was used.^[1]^ Indexing was performed by iterative use of singular value decomposition (LSI),^[2]^ leading to a triclinic unit cell with lattice parameters given in Table 1 in the main article. The most probable space group was determined to be *P*$\mathop{1}\limits^{\_}$ (2). The number of formula units per unit cell was estimated to *Z* = 2 from volume increments. The peak profiles and precise lattice parameters of the powder pattern were determined by a Pawley fit,^[3]^ using the fundamental parameter (FP) approach.^[4]^ For modeling of the background, Chebychev polynomials of higher order were employed. The refinement converged quickly. The structure determination was performed in the space group *P*$\mathop{1}\limits^{\_}$ by the method of Simulated Annealing (SA).^[5]^ The oxalate group and the phosphate anion were introduced as rigid bodies with flexible bond lengths but fixed angles. The positions of the atoms were found iteratively in SA runs. The crystal structure was validated by Rietveld refinement (Fig. S6).^[6]^ The thermal displacement factors were freely refined and the agreement factors are listed in Table 1.

**X-ray Photoelectron Spectra (XPS) studies**

XPS spectra of rGO/[K_2_(VO)_2_(HPO_4_)_2_(C_2_O_4_)] composite is shown in Fig. 2d-f in the main article. The peaks at binding energies of 293.14(±0.2) and 295.91(±0.2) eV, which correspond to K2p_3/2_ and K2p_1/2_ energy levels respectively due to spin orbit coupling (Fig. 2e). The C1s peaks due to the C_2_O_4_ group appeared at 289.03(±0.2) and 285.91(±0.2) eV while the C1s peaks due to the rGO layers appeared at 284.80(±0.2) eV. The spectrum of P2p is shown in Fig. 2f with Binding energy of 133.84(±0.2) eV, characteristic of tetrahedral PO_4_ group. The spectrum of O1s shown in Fig. 2d has binding energy of 532.04(±0.2) eV while the binding energies (BE) values of V2p are 517.30(±0.2) and 524.69 (±0.2) eV, which correspond to energy levels V2p_3/2_ and V2p_1/2_ respectively. The binding energy values of the different elements match with the reported values.^[7]^

Raman spectroscopy

Raman spectroscopy of the rGO/ of K_2_[(VO)_2_(HPO_4_)_2_(C_2_O_4_)]∙4.5H_2_O was carried out to analyse the presence of rGO in the composites. Fig. S9 shows the Raman spectra of bare GO sample and rGO composites of K_2_[(VO)_2_(HPO_4_)_2_(C_2_O_4_)]∙4.5H_2_O containing 4 and 8 % rGO. Bare GO (Fig. S9) shows Raman bands at 1600 and 1350 cm^-1^ which are described as G and D bands respectively. The D band is attributed to the defects and disordered atomic arrangement caused by sp^3^-carbon atom while the plane vibration of the sp^2^-carbon atom in the two dimensional lattice gives rise to the G band. The I_D_/I_G_ ratio of Graphene oxide is lower, while the reduction of GO results in higher I_D_/I_G_ ratio. In the case of the rGO composites, the I_D_/I_G_ ratio are higher than the bare GO indicating the reduction of graphene oxide. The composite with 4 % rGO shows I_D_/I_G_ ratio of close to unity, indicating better reduction of GO in the sample.

References

[1] Bruker AXS, Topas, version 4.1. 2007.

[2] Coelho, A. A. *J. Appl. Cryst*, 2003, *36*, 86-95.

[3] Pawley, G. S. *J. Appl. Cryst.* **1981**, *14*, 357.

[4] Cheary, R. W.; Coelho, A. A.; Cline,J. P. *J. Res. Natl. Inst. Stand. Technol.* 2005, *109*, 1-25.

[5] Andreev, Y. G.; MacGlashan, G. S.; Bruce, P. G. *Phys. Rev. B* 1997, *55*, 12011.

[6] Rietveld, H. M. *J. Appl. Crystallogr*. **1969**, *2*, 65-71.

[7] Nagarathinam, M., Saravanan, K., Phua, E. J. H., Reddy, M. V., Chowdari, B. V. R., Vittal J. J. *Angew. Chem. Int. Ed.* **2012**, 51, 5866-5870.
